# Phototropin 1 Mediates High-Intensity Blue Light-Induced Chloroplast Accumulation Response in a Root Phototropism 2-Dependent Manner in *Arabidopsis phot2* Mutant Plants

**DOI:** 10.3389/fpls.2021.704618

**Published:** 2021-09-27

**Authors:** Jing Wang, Yu-ping Liang, Jin-dong Zhu, Yu-xi Wang, Meng-ya Yang, Hong-ru Yan, Qian-yi Lv, Kai Cheng, Xiang Zhao, Xiao Zhang

**Affiliations:** State Key Laboratory of Crop Stress Adaptation and Improvement, State Key Laboratory of Cotton Biology, School of Life Sciences, Henan University, Kaifeng, China

**Keywords:** RPT2, phot1, high-intensity blue light, chloroplast accumulation, phot2

## Abstract

Phototropins, namely, phototropin 1 (phot1) and phototropin 2 (phot2), mediate chloroplast movement to maximize photosynthetic efficiency and prevent photodamage in plants. Phot1 primarily functions in chloroplast accumulation process, whereas phot2 mediates both chloroplast avoidance and accumulation responses. The avoidance response of phot2-mediated chloroplasts under high-intensity blue light (HBL) limited the understanding of the function of phot1 in the chloroplast accumulation process at the HBL condition. In this study, we showed that the phot2 mutant exhibits a chloroplast accumulation response under HBL, which is defective when the root phototropism 2 (RPT2) gene is mutated in the phot2 background, mimicking the phenotype of the phot1 phot2 double mutant. A further analysis revealed that the expression of RPT2 was induced by HBL and the overexpression of RPT2 could partially enhance the chloroplast accumulation response under HBL. These results confirmed that RPT2 also participates in regulating the phot1-mediated chloroplast accumulation response under HBL. In contrast, RPT2 functions redundantly with neural retina leucine zipper (NRL) protein for chloroplast movement 1 (NCH1) under low-light irradiation. In addition, no chloroplast accumulation response was detected in the *phot2 jac1* double mutant under HBL, which has been previously observed in *phot2 rpt2* and *phot1 phot2* double mutants. Taken together, our results indicated that phot1 mediates the HBL-induced chloroplast accumulation response in an RPT2-dependent manner and is also regulated by j-domain protein required for chloroplast accumulation response 1 (JAC1).

## Key Message

We demonstrate the role of RPT2 in regulating phot1-mediated chloroplast accumulation under HBL.

## Introduction

Chloroplast photorelocation is a critical physiological process that optimizes photosynthetic efficiency and ensures plant survival. To maximize light capture under low-intensity blue light (LBL), chloroplasts have been shown to accumulate toward the periclinal walls (Gotoh et al., 2018). In contrast, chloroplasts relocate toward the anticlinal walls as an avoidance response under high light conditions (Kasahara et al., 2002; Suetsugu and Wada, 2007; Kong et al., 2013). In most plants, chloroplast movement is primarily regulated by a blue light (BL) stimulus, although some cryptogam plants have been shown to exhibit a red light-mediated chloroplast movement response (Wada et al., 1993, 2003). Thus far, several key components that regulate BL-induced chloroplast movement have been identified, which include the BL receptors phototropin 1 (phot1) and phototropin 2 (phot2) (Suetsugu and Wada, 2007). *Arabidopsis* PHOT proteins, phot1 and phot2, each contain two light-oxygen-voltage (LOV) photosensory domains, LOV1 and LOV2, at the N-terminus and a serine/threonine kinase domain at the C-terminus (Hanks and Hunter, 1995; Huala et al., 1997; Christie et al., 1999). The LOV1 domain primarily regulates phototropin di/multimerization (Salomon et al., 2004; Nakasako et al., 2008; Nakasone et al., 2013), while LOV2 mainly regulates the C-terminal kinase domain through a BL-induced derepression (Christie et al., 2002; Harper et al., 2003; Jones et al., 2007; Jones and Christie, 2008; Nakasako et al., 2008; Tokutomi et al., 2008). In the dark, LOV2 binds with the kinase domain and inhibits its phosphorylation activity (Christie et al., 1999). Upon the absorption of light, the binding of LOV2 to the kinase domain is inhibited, resulting in the activation of kinase activity (Christie et al., 1999; Salomon et al., 2000; Inoue et al., 2008; Sullivan et al., 2008). Although both phot1 and phot2 mediated chloroplast movement response, the abundance and phosphorylation levels of phototropins and the interactions between phototropin molecules and phototropin homo and heterocomplexes vary under different light conditions in regulating chloroplast movement (Sztatelman et al., 2016). For example, phot1 and phot2 play different roles in the chloroplast accumulation response in *Arabidopsis*, but the avoidance response is controlled solely by phot2 under bright white light irradiation (Kagawa et al., 2001; Luesse et al., 2010; Ishishita et al., 2020). Consistent with this result, we have also previously shown that phot2 plays a role in chloroplast avoidance in *Gossypium hirsutum* (Shang et al., 2019).

In addition to two BL photoreceptors, chloroplast actin (cp-actin) filaments have been reported to participate in the chloroplast movement response (Kadota et al., 2009). BL regulates the relocation of cp-actin filaments to the front edge of chloroplasts during the chloroplast photorelocation process (Kadota et al., 2009). In addition, the abundance of cp-actin filaments between the leading and retral portion of chloroplasts rises with increasing light intensity, suggesting that light plays an important role in the determination of the relocalization of cp-actin filaments, thereby regulating the speed of chloroplast avoidance (Kadota et al., 2009; Suetsugu et al., 2010). The localization of cp-actin filaments at the interface between the chloroplast and the plasma membrane depends primarily on chloroplast unusual positioning 1 (CHUP1), kinesin-like protein for actin-based chloroplast movement 1 (KAC1), and kinesin-like protein for actin-based chloroplast movement 2 (KAC2) (Kadota et al., 2009; Suetsugu et al., 2010, 2016a). A previous study revealed that the KAC protein increases cp-actin filament abundance and the speed of chloroplast avoidance movement (Suetsugu et al., 2010). In addition, weak chloroplast movement under BL1 (WEB1) and plastid movement impaired 2 (PMI2) also control the speed of chloroplast photorelocation by regulating the abundance of cp-actin filaments in *Arabidopsis* (Luesse et al., 2006; Kodama et al., 2010).

Chloroplast accumulation and avoidance responses are regulated by different signals, which have been demonstrated through observations showing different initial avoidance and accumulation movements in *Lemna trisulca* (Malec and Murchadha, 1994). Distinct chloroplast movement behavior was also observed upon light irradiation in *Adiantum capillus-veneris* (Kagawa and Wada, 1999). The signal for the accumulation response is sustained over long durations, whereas the one for the avoidance response persists for a shorter period of time and gets weaker and weaker quickly when the light is turned off (Kagawa and Wada, 1999). Root phototropism 2 (RPT2) and nonphototropic hypocotyl 3 (NPH3) are key regulators of leaf orientation, flatness, and hypocotyl phototropism, but not stomatal movement (Inada et al., 2004; Inoue et al., 2008; Tsutsumi et al., 2013). Neural retina leucine zipper (NRL) protein for chloroplast movement 1 (NCH1) and RPT2 have also been shown to be necessary for phototropin-dependent chloroplast accumulation responses (Suetsugu et al., 2016b). However, NPH3, RPT2, and NCH1 do not participate in the avoidance response (Suetsugu et al., 2016b), indicating their specificity in regulating chloroplast movement. Despite these earlier findings, the actual mechanism that underlies this specificity is unknown.

To determine which components differentiate the accumulation and avoidance responses, we used RPT2 as a bait to identify its interacting proteins using yeast two-hybrid screening. Six proteins, including j-domain protein required for chloroplast accumulation response 1 (JAC1), were isolated (Zhao et al., 2018). The protein JAC1 has been characterized as an important factor for phototropin-regulated chloroplast accumulation and phot2-dependent dark positioning (Suetsugu et al., 2005). However, JAC1 is unnecessary for the avoidance response mediated by phot2 under high light irradiation (Suetsugu et al., 2005). In this study, we employed a white and green band (WGB) assay to analyze chloroplast movement responses based on changes in leaf transmittance. Briefly, a white band (WB) in rosette leaves indicates a chloroplast avoidance response, whereas a green band (GB) indicates a chloroplast accumulation response.

We found that similar to the *phot1 phot2* and *phot2 jac1* double mutants, the *rpt2 phot2* double mutant exhibited significantly reduced chloroplast accumulation under high-intensity blue light (HBL) (100 μmol m^−2^ s^−1^). We also found that the mutation of *RPT2* attenuated the chloroplast accumulation response in *phot1* mutants under intermediate-intensity BL (IBL) (5 μmol m^−2^ s^−1^) and LBL (.01–1 μmol m^−2^ s^−1^). In addition, the overexpression of *RPT2* partially enhanced chloroplast accumulation under HBL. These results indicated that RPT2 participates in phot1-mediated-chloroplast accumulation under HBL irradiation, which differs from the published data showing that RPT2 functioned redundantly with NCH1 to regulate chloroplast accumulation movement in response to LBL (Suetsugu et al., 2016b). A further study indicated that JAC1 also functions downstream of RPT2 to mediate chloroplast accumulation under HBL irradiation.

## Materials and Methods

### Plant Material and Growth Condition

The mutants *phot1-5, phot2-1*, and *phot1-5 phot2-1* were supplied by Dr. Ken-ichiro Shimazaki (Kyushu University, Japan) (Kinoshita et al., 2001), and the *rpt2, nph3-6, rpt2 phot1, phot1 nph3-6, rpt2 phot2*, and *phot2 nph3-6* mutants were obtained from by Dr. Christian Fankhauser (University of Lausanne, Switzerland). Additionally, the *cry1 cry2* and *phot1 phot2 cry1 cry2* mutants were provided by Professor Hong-quan Yang. The mutant *jac1* (SAIL_574_B09) was purchased from the *Arabidopsis* Biological Resource Center (ABRC), and the *rpt2 jac1, jac1 phot1*, and *jac1 phot2* mutants were generated in this study by crossing. For crosses, *jac1* was used as the female parent and crossed with *phot1, phot2*, and *rpt2*. Homozygous double mutants were selected from the F2 populations. The coding sequence of RPT2 was fused to the N-terminal of GFP in the pCAMBIA1300 vector (Zhao et al., 2015). The constructs were introduced into the *Agrobacterium tumefaciens* strain GV3101 and transformed by floral infiltration into *Arabidopsis* wild-type (WT) plant (col-0) and *phot2*. Transformants with a 3:1 segregation ratio were self-pollinated, and the homozygous progeny resistant to 25 μg/ml of hygromycin B was selected. All seeds were surface-sterilized with 0.1% mercuric chloride for 5 min, washed five times with sterile water, and then sown on Murashige and Skoog media that contained 3% sucrose and 0.6% agar. Sterilized seeds were placed in the dark at 4°C for 3 days and then transferred to a cultivation room under white light (70 μmol m^−2^ s^−1^) at 22°C and 16-h light /8-h dark conditions for 7 days and then transferred to soil. Plants were grown for 3 weeks for chloroplast movement analyses.

### Chloroplast Photorelocation Movement Assays

Plants were grown normally in soil for 3 weeks. Rosette leaves were then detached and pretreated with darkness for 12 h. Leaves were then placed on a 0.8% agar medium, covered with a sheet of foil paper with a 2-mm wide nonopaque slit, and exposed to continuous vertical irradiation with 1, 5, or 100 μmol m^−2^ s^−1^ of BL by using light-emitting diode BL lamps. The fluence rate was controlled by filters (film no. 72; Tokyo Butai Shoumei). After irradiation, chloroplast movement was analyzed using a WGB assay, where rosette leaves exhibiting a WB indicated a chloroplast avoidance response and those with a GB indicated a chloroplast accumulation response (Kagawa et al., 2001). Light transmittance through leaf tissues was measured with a Epoch2 photosensor (BioTek, America). Leaf transmittance assays were carried out as described in the previous study (Kodama et al., 2010).

### Gene Expression Analysis

A ribonucleic acid extraction kit (RK101-02, Lifefeng, Shanghai in China) was used to extract the total RNA from 3-week-old rosette leaves irradiated with 0, 1, 5, or 100 μmol·m^−2^·s^−1^ of BL. Reverse transcription was performed using 1 μg of total RNA *via* a Hiscript®III RT SuperMix for qPCR (R323-01, Vazyme Biotech Co., Ltd. Nanjing in China). The resulting cDNA was diluted 10-fold and used as a template for Quantitative Real-time PCR (qRT-PCR). qRT-PCR assays were performed using a ChamQ Universal SYBR qPCR Master Mix kit (Vazyme, nanjing) on a Roche480 fluorescence qPCR instrument. ACTIN2 was used as the internal control for quantitative analysis, and other genes were amplified using gene-specific primer pairs (Supplementary Table S1).

### Immunodetection

Total protein was extracted from the 3-week-old rosette leaves of transgenic plants expressing P35S:RPT2-GFP in the phot2-1 mutant background with a lysis buffer [100 mM of TRIS hydrochloride (Tris-HCl), pH 6.8, 10% 2-mercaptethanol, 4% Sodium dodecyl sulfate (SDS), 20% glycerol, 5 mM of dithiothreitol (DTT), and 100 μM of Phenylmethylsulfonyl fluoride (PMSF)] and protein concentration was quantified using the Quick Start Bradford 1xDye Reagent (Bio-Rad), according to the protocol of the manufacturer. Afterward, 10 μg of protein was separated on 10% sodium dodecyl sulfate–polyacrylamide gel electrophoresis (SDS-PAGE) gels. Western blot experiments were carried out as previously described (Zhao et al., 2013). Anti- green fluorescent protein (GFP) antibodies (Abmart, MA9023) were used at a dilution of 1:2,000, and anti-actin antibodies (Abbkin, A01050) were used at a dilution of 1:3,000. The secondary antibody [goat anti-rabbit horseradish peroxidase (HRP)-conjugated immunoglobulin G (IgG), Abmart, M21002] was used at a dilution of 1:5,000. The signal was detected with a Fusion FX6-XT (VILBER). The ACTIN protein was used as the loading control.

## Results

### RPT2 Regulates phot1-Mediated Chloroplast Accumulation Under HBL Irradiation

Both phot1 and phot2 have been shown to be functionally redundant in the regulation of chloroplast accumulation under LBL (0.01–1 μmol m^−2^ s^−1^), whereas only phot1 is involved in chloroplast accumulation under HBL (100 μmol m^−2^ s^−1^) (Sakai et al., 2001).

**Figure 1:**
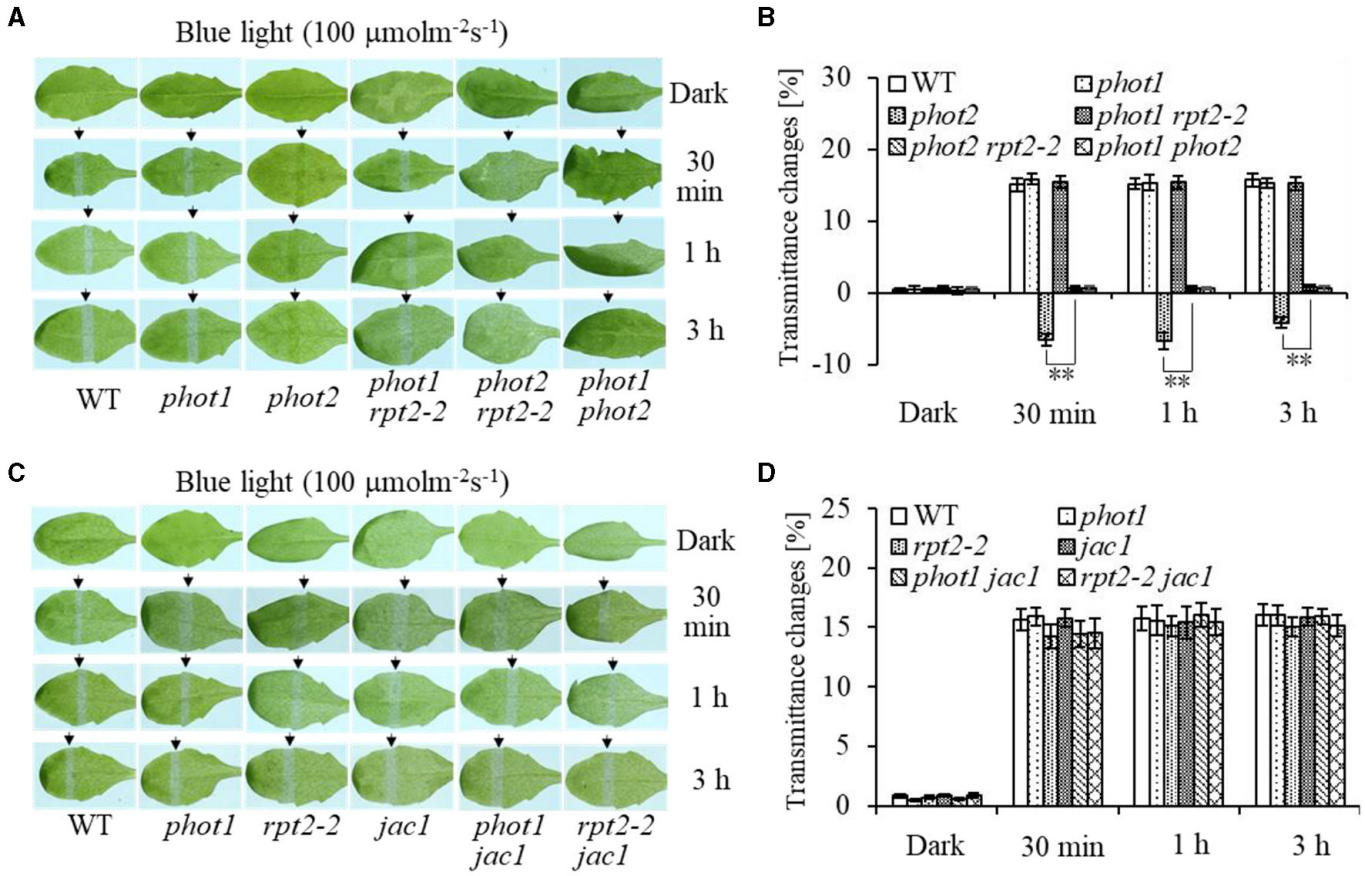
Chloroplast movements in response to high-intensity blue light (HBL) in wild-type (WT) and different mutants. Detached 21-day-old rosette leaves pretreated with 12 h of darkness were placed on a 0.8% agar medium, covered with a light barrier with a 2-mm wide nonopaque slit, and irradiated with 100 μmol m^−2^ s^−1^ of continuous blue light (BL) (**A**: WT, *phot1, phot2, phot1 rpt2, phot2 rpt2*, and *phot1 phot2*; **C**: WT, *phot1, rpt2-2, jac1, phot1 jac1*, and *rpt2-2 jac1*) for 30 min, 1 h, or 3 h in a growth chamber. The arrow indicates that the BL irradiation direction was perpendicular to the leaves. Light transmittance was measured in rosette leaves (**B**: WT, *phot1, phot2, phot1 rpt2, phot2 rpt2*, and *phot1 phot2*; **D**: WT, *phot1, rpt2-2, jac1, phot1 jac1*, and *rpt2-2 jac1*). Error bars represent the *SD* of 21 rosette leaves. ^**^p <.01 (Student's *t*-test); ns denotes no significant differences between groups.

To investigate the function of phot1 in chloroplast accumulation under HBL irradiation, chloroplast photorelocation movements were analyzed in different mutants. Under HBL (100 μmol m^−2^ s^−1^), the *phot1* mutant showed no significant difference in chloroplast avoidance compared with the wild type, whereas the *phot2* mutant showed chloroplast accumulation and the *phot1 phot2* double mutant was deficient in both chloroplast avoidance and accumulation responses (Figures 1A,B). Notably, the chloroplast accumulation response of the *phot2* mutant under HBL irradiation was inhibited by the mutation of the *RPT2* gene, mimicking the phenotype of the *phot1 phot2* double mutant (Figures 1A,B). However, the phenotypes of the *rpt2, jac1, phot1 rpt2, phot1 jac1*, and *rpt2 jac1* mutants were similar to those of *phot1* or the WT plants under HBL irradiation (Figure 1). These results indicated that the phot1-mediated chloroplast accumulation response was mainly dependent on RPT2 under HBL irradiation.

We also examined the contribution of NPH3 and cryptochromes to chloroplast movement and found that mutations in the *NPH3* gene did not affect chloroplast movement under all fluence rates of BL (Supplementary Figure S1), which is consistent with published data (Suetsugu et al., 2016b). To determine whether cryptochromes play a role in chloroplast photorelocation, the chloroplast movement response of the *cry1 cry2* double mutant was analyzed in *Arabidopsis* rosette leaves irradiated with 3 and 100 μmol m^−2^ s^−1^ of BL. This experiment revealed that the chloroplast movement response in the *cry1 cry2* double mutant was indistinguishable from the WT (data not shown).

### JAC1 Regulates RPT2-Mediated Chloroplast Accumulation Under HBL Irradiation

**Figure 2:**
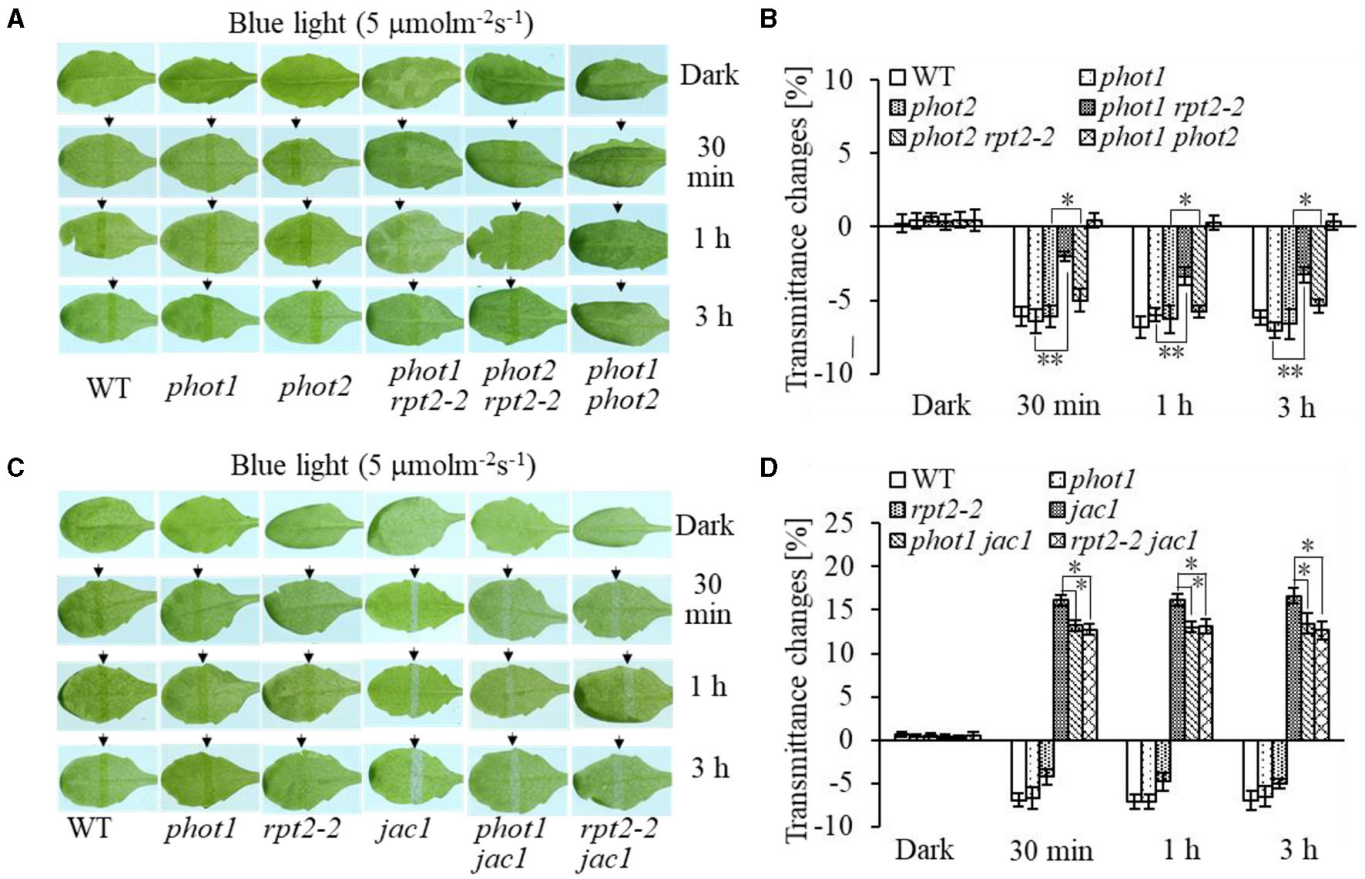
Chloroplast movements in response to intermediate-intensity blue light (IBL) in WT and different mutants. Detached 21-day-old rosette leaves pretreated with 12 h of darkness were placed on a 0.8% agar medium, covered with a light barrier with a 2-mm wide nonopaque slit, and irradiated with 5 μmol m^−2^ s^−1^ of continuous BL (**A**: WT, *phot1, phot2, phot1 rpt2, phot2 rpt2*, and *phot1 phot2*; **C**: WT, *phot1, rpt2-2, jac1, phot1 jac1*, and *rpt2-2 jac1*) for 30 min, 1 h, or 3 h in a growth chamber. The arrow indicates that the BL irradiation direction was perpendicular to the leaves. Light transmittance was measured in rosette leaves (**B**: WT, *phot1, phot2, phot1 rpt2, phot2 rpt2*, and *phot1 phot2*; **D**: WT, *phot1, rpt2-2, jac1, phot1 jac1*, and *rpt2-2 jac1*). Error bars represent the *SD* of 21 rosette leaves. **p < 0.01; *p < 0.05 (Student's *t*-test); ns denotes no significant differences between groups.

**Figure 3:**
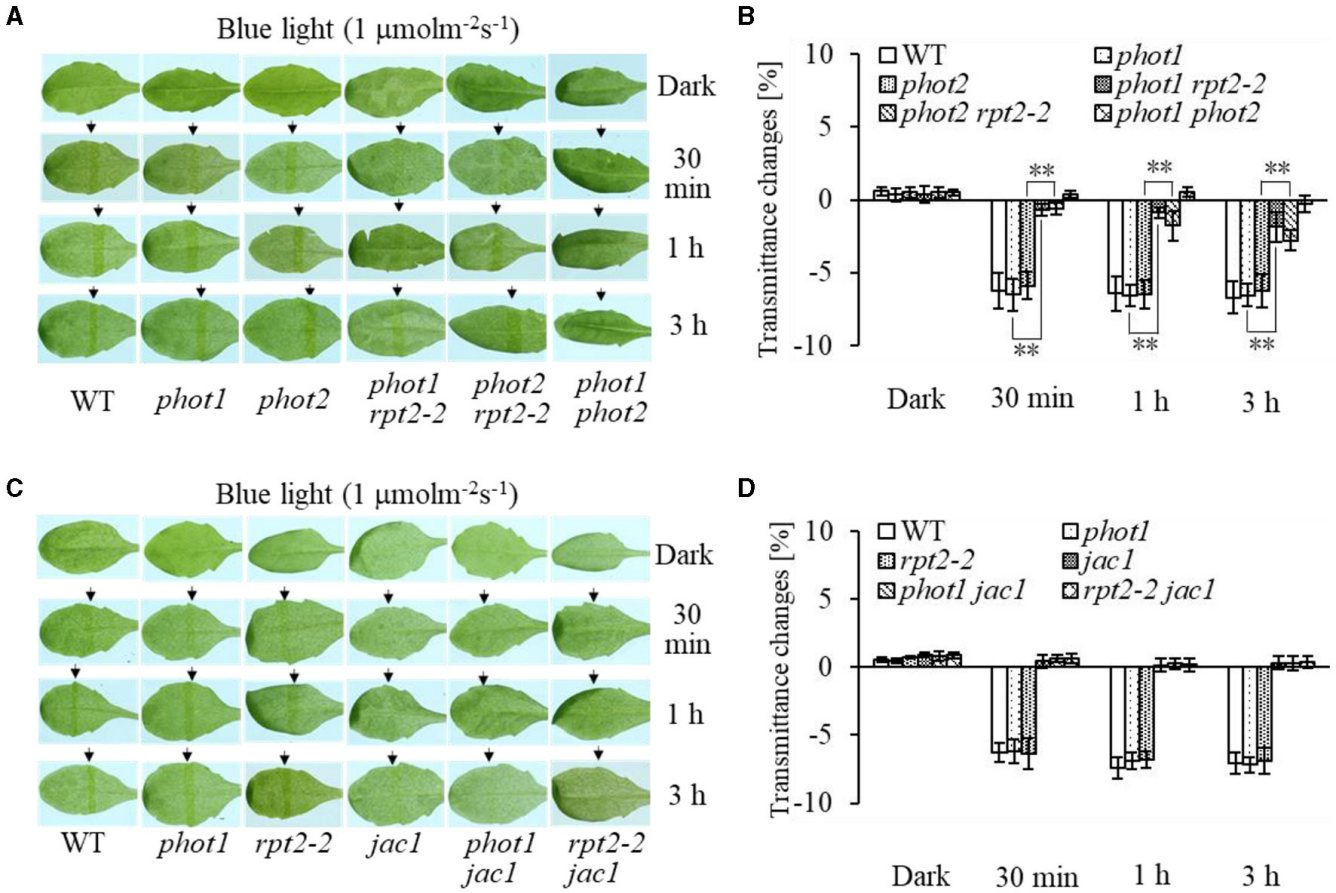
Chloroplast movements in response to low-intensity blue light (LBL) in WT and different mutants. Detached 21-day-old rosette leaves pretreated with 12 h of darkness were placed on a 0.8% agar medium, covered with a light barrier with a 2-mm wide nonopaque slit, and irradiated with 1 μmol m^−2^ s^−1^ of continuous BL (**A**: WT, *phot1, phot2, phot1 rpt2, phot2 rpt2*, and *phot1 phot2*; **C**: WT, *phot1, rpt2-2, jac1, phot1 jac1*, and *rpt2-2 jac1*) for 30 min, 1 h, or 3 h in a growth chamber. The arrow indicates that the BL irradiation direction was perpendicular to the leaves. Light transmittance was measured in rosette leaves (**B**: WT, *phot1, phot2, phot1 rpt2, phot2 rpt2*, and *phot1 phot2*; **D**: WT, *phot1, rpt2-2, jac1, phot1 jac1*, and *rpt2-2 jac1*). Error bars represent the *SD* of 21 rosette leaves. **p < 0.01 (Student's *t*-test).

Chloroplast accumulation movement has been reported to be regulated redundantly by phot1 and phot2 under LBL irradiation, whereas the chloroplast avoidance response is mediated by phot2 alone (Sakai et al., 2001; Inada et al., 2004, de Carbonnel et al., 2010; Sztatelman et al., 2016; Shang et al., 2019). In addition, RPT2 functions redundantly with NCH1 to regulate chloroplast accumulation movement in response to LBL (Suetsugu et al., 2016b). To investigate the role of RPT2 in chloroplast accumulation under LBL or IBL and determine the relationship between RPT2 and phot2, we analyzed the chloroplast accumulation under LBL or IBL in different mutants. There was no obvious difference in the chloroplast accumulation response in *phot1* or *phot2* under LBL (1 μmol m^−2^ s^−1^) or IBL (5 μmol m^−2^ s^−1^) irradiation compared with WT (Figures 2A,B, **3A,B**). However, the mutation of *RPT2* significantly reduced the chloroplast accumulation in the *phot1*, but not *phot2*, background (Figures 2A,B, **3A,B**). The *phot1 rpt2* double mutant exhibited a weaker chloroplast accumulation response compared with the *phot2 rpt2* double mutant, indicating that phot2-mediated chloroplast accumulation is mainly dependent on RPT2 under LBL and IBL.

**Figure 4:**
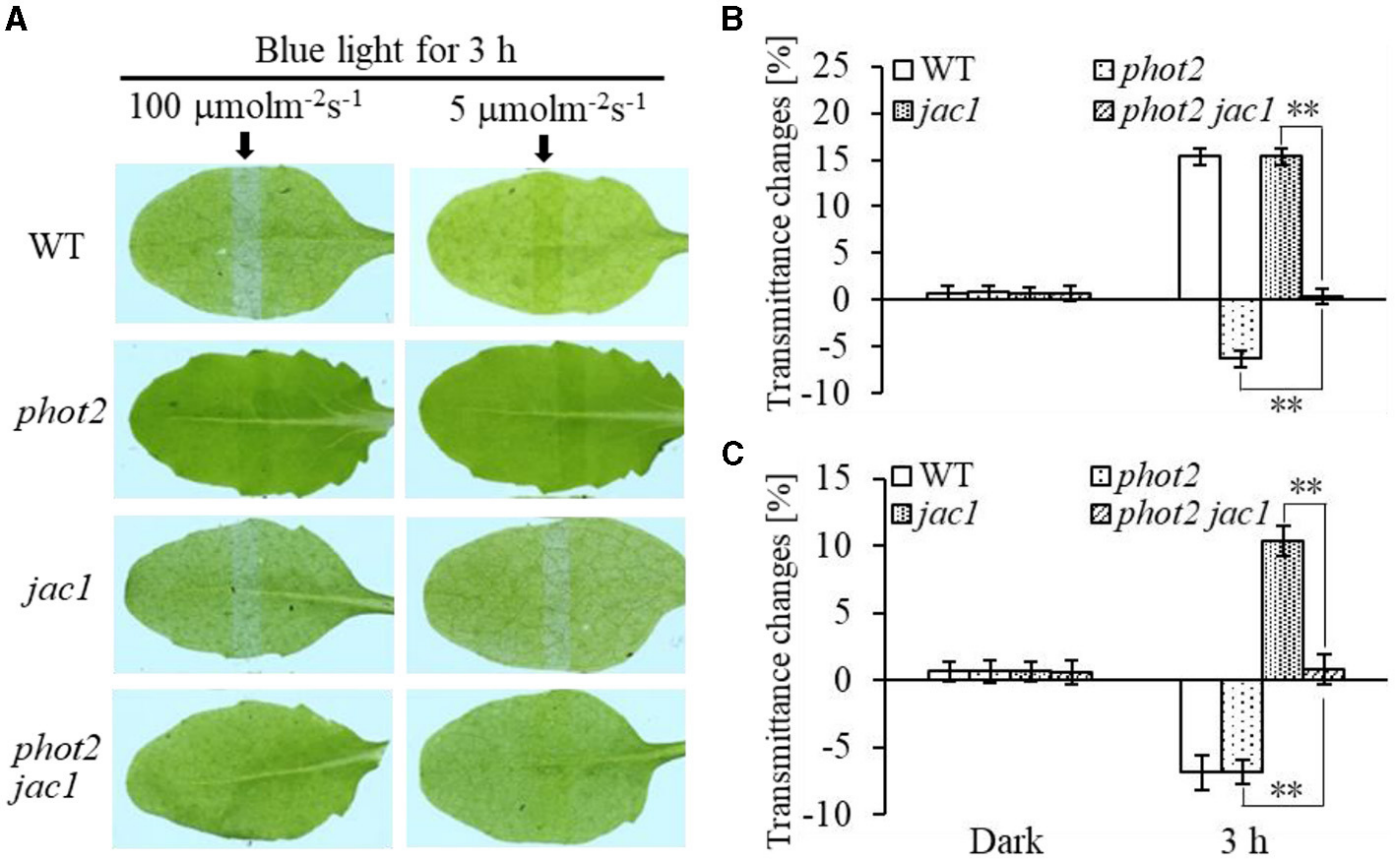
Chloroplast movements in response to different intensities of blue light in WT, *phot2, jac1*, and *phot2 jac1*. **(A)** Detached 21-day-old rosette leaves of WT and mutant plants pretreated with 12 h of darkness were placed on a 0.8% agar medium, covered with a light barrier with a 2-mm wide nonopaque slit, and irradiated with continuous BL (Left: 100 μmol m^−2^ s^−1^, Right: 5 μmol m^−2^ s^−1^) for 3 h in a growth chamber. The arrow indicates that the BL irradiation direction was perpendicular to the leaves. Light transmittance was measured in rosette leaves (**B**: 100 μmol m^−2^ s^−1^, **C**: 5 μmol m^−2^ s^−1^). Error bars represent the *SD* of 21 rosette leaves. **p < 0.01 (Student's *t*-test).

Furthermore, we analyzed chloroplast movements in *jac1, phot1 jac1*, and *rpt2 jac1* and found that *jac1* mutant plants exhibited chloroplast avoidance movements in response to IBL (5 μmol m^−2^ s^−1^) (Figures 2C,D). Both *phot1 jac1* and *rpt2 jac1* double mutants also exhibited chloroplast avoidance responses under IBL, although these responses were weaker than those of the *jac1* single mutant (Figures 2C,D). However, *jac1, phot1 jac1*, and *rpt2 jac1* exhibited a defect in their chloroplast avoidance and accumulation responses under LBL (1 μmol m^−2^ s^−1^) when compared with wt, *phot1*, and *rpt2* (Figures 3C,D). Interestingly, the chloroplast accumulation response of the *phot2* mutant under HBL irradiation was inhibited by mutating the *JAC1* gene (Figures 4A,B), which resulted in a phenotype similar to that of the *phot2 rpt2* double mutant (Figures 1A,B). In addition, no chloroplast accumulation or avoidance response was detected under either of the HBL (100 μmol m^−2^ s^−1^) or IBL (5 μmol m^−2^ s^−1^) conditions in the *phot2 jac1* double mutants (Figure 4). These data, together with the finding that the *jac1* mutant was deficient in chloroplast accumulation under LBL (1 μmol m^−2^ s^−1^), suggested that JAC1 is essential for chloroplast accumulation at all rates of BL. Given the similar phenotypes of *jac1* and *rpt2 jac1* under IBL and HBL (Figures 1, 2C,D), we propose that JAC1 functions downstream of RPT2 alter phot1-mediated chloroplast accumulation under HBL irradiation.

### Contribution of Cryptochromes to Changes in Gene Expression in Response to BL

**Figure 5:**
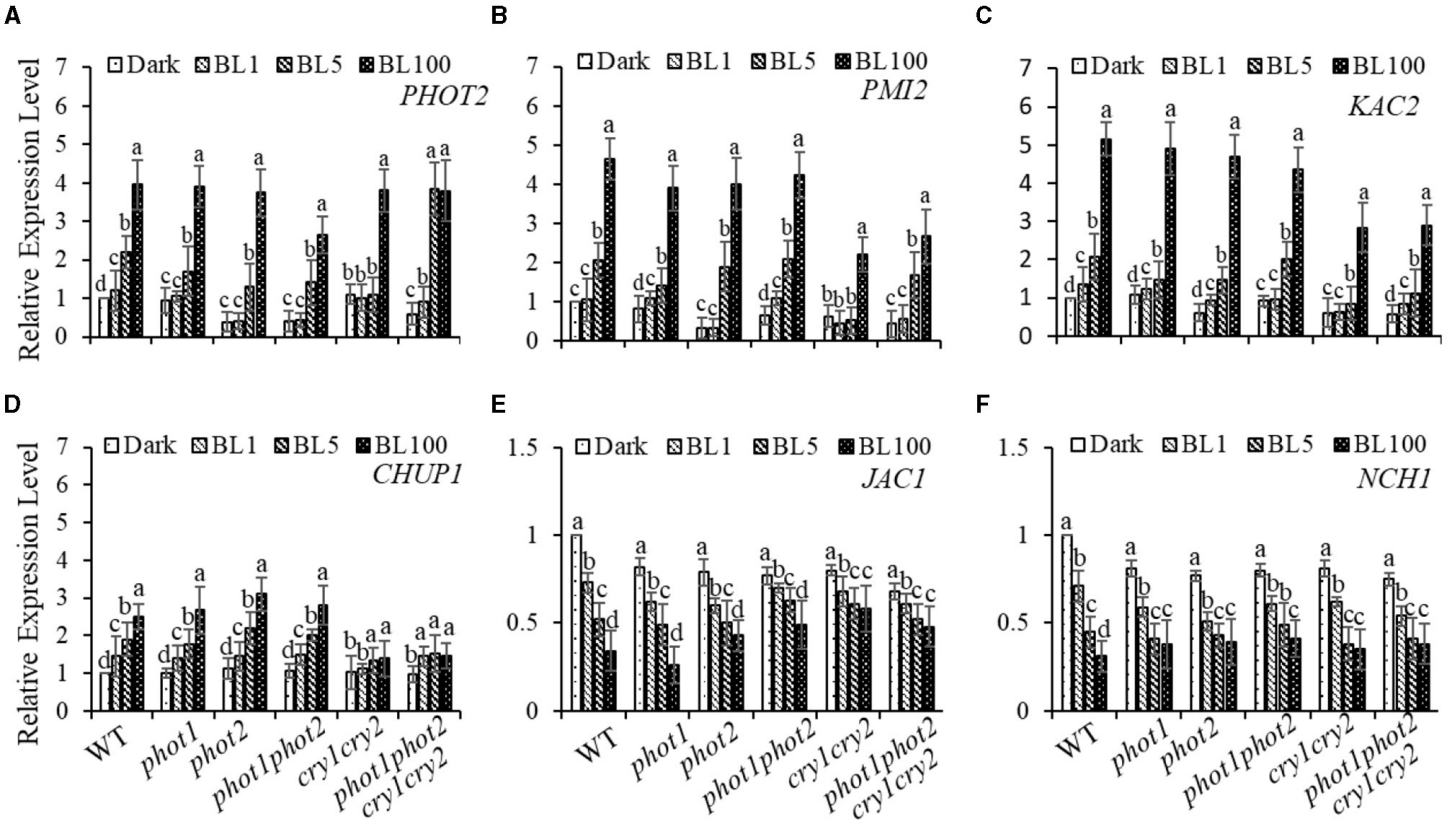
Quantitative RT-PCR analysis of the expression of chloroplast-movement-related genes. The 21-day-old rosette leaves of WT, *phot1, phot2, phot 1phot2, cry1 cry2*, or *phot1 phot2 cry1 cry2* were exposed to continuous BL (BL1 = 1 μmol m^−2^ s^−1^, BL5 = 5 μmol m^−2^ s^−1^, BL100 = 100 μmol m^−2^ s^−1^) for 3 h, and the transcript levels of *PHOT2* **(A)**, *PMI2* **(B)**, *KAC2* **(C)**, *CHUP1* **(D)**, *JAC1* **(E)**, and *NCH1* **(F)** were detected. The value of dark-treated WT was set to 1. Error bars show the *SD* of three independent biological replicates (*n* = 3). A one-way ANOVA and Tukey's tests were performed to identify significant differences. Groups marked with different letters are significantly different (*p* < 0.05).

To further explore the impacts of different BL receptors on chloroplast movement regulation, we assessed the expression of genes that have been implicated in chloroplast avoidance movement, including *PHOT2, PMI2*, and *CHUP1* (Kasahara et al., 2002; Oikawa et al., 2003). Consistent with our hypothesis, the expression levels of *PHOT2* and *PMI2* were positively correlated with increases in BL irradiation (Figures 5A,B). Moreover, we found that the BL-induced expression of *CHUP1* is partly blocked in the *cry1 cry2* and *phot1 phot2 cry1 cry2* (Figure 5D) mutants. Furthermore, *kac1kac2* is deficient in its avoidance and accumulation responses and has chloroplasts detached from the plasma membrane, which indicates that KAC1 and KAC2 regulate chloroplast movement and anchor to the plasma membrane (Suetsugu et al., 2010). Consistently, we found that the expression of *KAC1*and *KAC2* is induced obviously by HBL, even in the *cry1 cry2* and *phot1 phot2 cry1 cry2* mutants (Figure 5C and Supplementary Figure S2B). These results supported the involvement of gene re-induced expression in the mediation of chloroplast avoidance movement in response to HBL, with this process possibly not being dependent on cryptochromes. In addition, the expression of *JAC1* and *NCH1*, which are involved in chloroplast accumulation movement, was partly inhibited with increasing intensity of BL irradiation (Figures 5E,F), similar to previously reported results (Suetsugu et al., 2016b). WEB1 has been shown to play a key role in the determination of the speed of chloroplast movements through the regulation of cp-actin filament amounts (Kodama et al., 2010). Therefore, we assessed the expression of *WEB1* in different BL receptor mutants in response to different intensities of BL and found that the expression of *WEB1* was not significantly different compared with WT (Supplementary Figure S2C).

### RPT2 Expression Impacts HBL-Induced Chloroplast Accumulation

**Figure 6:**
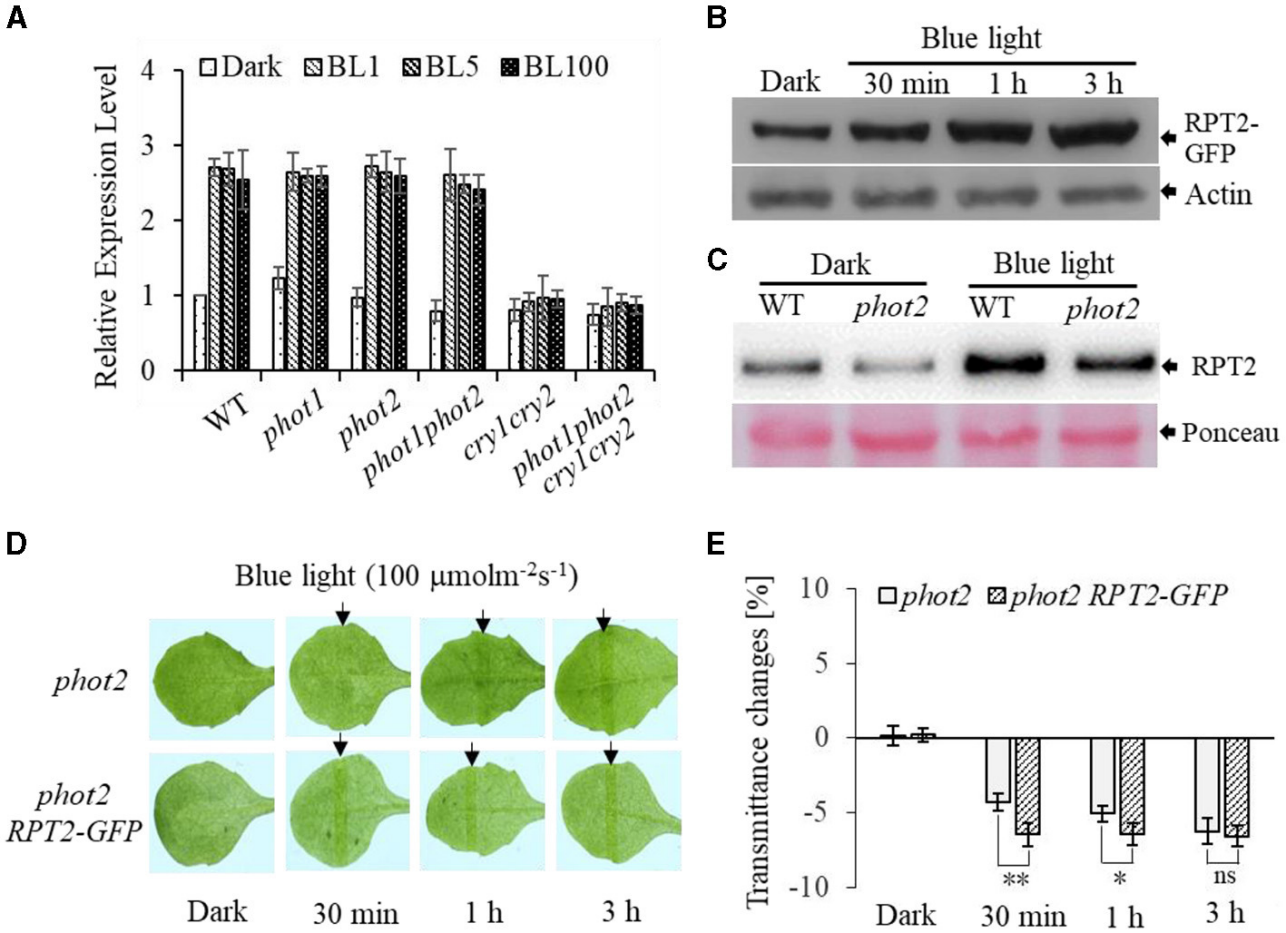
Root phototropism protein 2 (RPT2)-modulated high-intensity blue light (HBL)-induced chloroplast accumulation *via* transcriptional and post-transcriptional regulation in the absence of *phot2*. **(A)** Quantitative RT-PCR analysis of the expression of *RPT2*. WT, *phot1, phot2, phot1 phot2, cry1 cry2*, and *phot1 phot2 cry1 cry2* seedlings were exposed to continuous BL (BL1 = 1 μmol m^−2^ s^−1^, BL5 = 5 μmol m^−2^ s^−1^, BL100 = 100 μmol m^−2^ s^−1^) for 3 h, and the transcript levels of *RPT2* were measured. The value of dark-treated WT plants was set to 1. Error bars show the *SD* of three independent biological replicates (*n* = 3). **(B,C)** An immunoblot analysis of the RPT2 protein abundance. After a dark pretreatment for 12 h, plants were kept in darkness or exposed to BL at an intensity of 100 μmol·m^−2^·s^−1^ and subjected to anti-GFP immunoprecipitation using anti-GFP antibodies **(B)** and anti-RPT2 immunoprecipitation using anti-RPT2 antibodies **(C)**. Actin and Ponceau staining served as the control. **(D)** Chloroplast movements in response to HBL in *phot2* and 35S:RPT2-GFP/*phot2* irradiated with 100 μmol m^−2^ s^−1^ of BL for 30 min, 1 h, or 3 h. **(E)** A quantitative analysis of leaf transmittance in *phot2* and 35S::RPT2-GFP/*phot2* irradiated with 100 μmol m^−2^ s^−1^ BL for 30 min, 1 h, or 3 h. Error bars represent the SD of 21 rosette leaves. **p < 0.01; *p < 0.05 (Student's *t-*test).

To further investigate the function of RPT2 in regulating chloroplast photorelocation movement, we first assessed the transcript level of *RPT2*. This analysis revealed that the RPT2 transcript level was significantly induced by BL irradiation in WT, *phot1, phot2*, and *phot1 phot2*, but not *cry1 cry2* or *phot1 phot2 cry1 cry2* (Figure 6A). Western blotting using polyclonal antisera against RPT2 confirmed that the expression of RPT2 protein was markedly induced by 3 h of BL irradiation in wild type and *phot2* mutants (Figure 6C). We also examined the abundance of RPT2 proteins in AtRPT2-GFP transgenic lines and found that BL significantly enhanced RPT2 protein levels in a time-dependent manner (Figure 6B). Under HBL irradiation, the overexpression of RPT2 in the homozygous *phot2* (*phot2 RPT2-GFP*) mutant enhanced chloroplast accumulation compared with *phot2* (Figures 6D,E), suggesting that the amount of RPT2 induced by HBL is vital for the regulation of chloroplast accumulation response. Collectively, these data demonstrated a key role of HBL-induced RPT2 in the regulation of phot1-mediated chloroplast accumulation in the absence of phot2.

## Discussion

**Figure 7:**
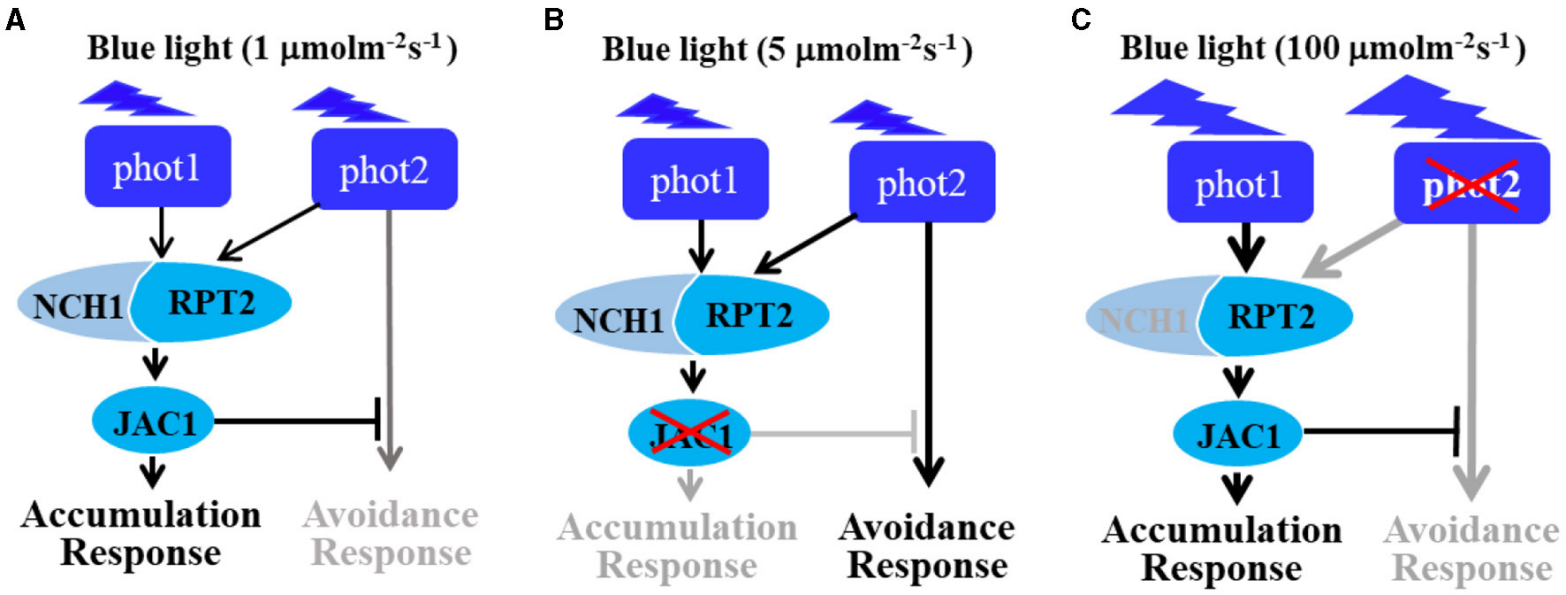
The role of RPT2 in mediating chloroplast movement response under continuous blue light. **(A)** RPT2, neural retina leucine zipper (NRL) protein for chloroplast movement 1 (NCH1), and j-domain protein required for chloroplast accumulation response 1 (JAC1) regulate the chloroplast accumulation responses mediated by phot1 and phot2 under LBL (1 μmol m^−2^ s^−1^). **(B)** Upon irradiation with continuous intermediate-IBL (5 μmol m^−2^ s^−1^), RPT2 partially contributes to chloroplast avoidance in the absence of JAC1. **(C)** Upon irradiation with continuous HBL (100 μmol m^−2^ s^−1^), the RPT2-mediated chloroplast accumulation response was mainly dependent on phot1 under HBL irradiation in the absence of phot2 (100 μmol·m^−2^·s^−1^). Gray arrows indicate the suppressed signaling pathways. Black arrows indicate the activated signaling pathways. The model was modified from a study by Suetsugu and Wada (2017).

Phototropins are BL receptor kinases that function redundantly to mediate chloroplast accumulation responses under LBL (0.01–1 μmol m^−2^ s^−1^) (Sakai et al., 2001). Phot2 is the only photoreceptor for the chloroplast avoidance response under HBL (100 μmol m^−2^ s^−1^) (Jarillo et al., 2001; Kagawa et al., 2001). At this light intensity, phot1 is also responsible for chloroplast accumulation (Sakai et al., 2001). Previous studies have mainly focused on LBL-induced chloroplast accumulation and HBL-induced avoidance responses (Kasahara et al., 2002; Suetsugu and Wada, 2007). However, the mechanisms underlying the avoidance response under LBL and accumulation response under HBL remained elusive. In this study, we provided evidence that both RPT2 and JAC1 regulate phot1-mediated chloroplast accumulation under HBL in the *Arabidopsis phot2* mutant (Figure 1). Our results are in agreement with previous reports indicating that RPT2, JAC1, and NCH1 all participate in the regulation of chloroplast accumulation, but the exact roles of these proteins appeared to be different in our study (Figure 7; Inada et al., 2004; de Carbonnel et al., 2010; Suetsugu et al., 2016b; Sztatelman et al., 2016). The *phot2* mutant showed chloroplast accumulation movement in response to HBL, while the lack of chloroplast accumulation in *phot2 rpt2* and *phot2 jac1*, mimicking the phenotype of the *phot1 phot2* double mutant, indicated that both RPT2 and JAC1 are necessary for a phot1-mediated accumulation response under HBL (Figures 1, 4). Under IBL, *rpt2* exhibited normal chloroplast accumulation, whereas *jac1* exhibited chloroplast avoidance (Figure 2). Under a lower light intensity, the *jac1* mutant lost both accumulation and avoidance responses, whereas the *rpt2* mutant exhibited a normal accumulation response (Figure 3). Based on these data, we proposed that JAC1 plays a positive role in chloroplast accumulation but a negative role in chloroplast avoidance. The avoidance response under HBL and chloroplast accumulation under IBL of the *rpt2* mutant, together with previous reports indicating that both NCH1 and RPT2 are necessary for phototropin-mediated chloroplast accumulation (Suetsugu et al., 2016b), indicated that RPT2 also positively regulates chloroplast accumulation under HBL. The *rpt2 nch1* double mutant is completely defective in the accumulation response (Suetsugu et al., 2016b), indicating that RPT2 functions redundantly with NCH1 under IBL and LBL. Moreover, the *jac1* and *jac1 rpt2* mutants exhibited severely impaired accumulation responses under weak BL (≤ 1 μmol m^−2^ s^−1^), whereas the *rpt2* mutant exhibited a normal accumulation response, suggesting that JAC1 functions downstream of RPT2 in regulating chloroplast accumulation (Suetsugu et al., 2005). These reports, together with the lack of chloroplast accumulation in the *phot2 rpt2* and *phot2 jac1* double mutants (Figure 1), indicated that JAC1 functions downstream of RPT2 to regulate phot1-mediated chloroplast accumulation under HBL.

Previously, the expression of *PHOT2* in mature leaves was shown to be significantly induced by high light irradiation (Labuz et al., 2012). We also found that *RPT2* expression significantly increased with increasing BL intensity (Zhao et al., 2020). Both KAC1 and PMI2 have been shown to be important for the regulation of chloroplast movement, although the underlying mechanisms of their functions are still unclear (Luesse et al., 2006; Suetsugu et al., 2010). CHUP1 regulates the chloroplast avoidance response (Oikawa et al., 2003, 2008; Braun and Schleiff, 2008) and functions in chloroplast photorelocation and attachment to the plasma membrane (Kasahara et al., 2002; Oikawa et al., 2003, 2008; Kadota et al., 2009). To explore the molecular mechanisms underlying chloroplast movement, we selected five genes for expression analysis: *PHOT2, PMI2, KAC2*, and *CHUP1* were induced by high light, whereas the expression levels of *JAC1* and *NCH1* decreased under high light (Figure 5). Although the expression of *RPT2* was lower under HBL than LBL, the expression was still induced significantly compared with dark conditions, which could explain LBL- and HBL-mediated chloroplast photorelocation movement. Immunoblotting also showed that RPT2 was induced by BL irradiation in a time-dependent manner (Figures 6B,C). We further demonstrated that the overexpression of *RPT2* strongly enhanced chloroplast accumulation in *phot2* under high light (Figures 6D,E), suggesting that HBL-induced RPT2 increases are vital for the regulation of chloroplast accumulation responses.

Based on the findings of this research and earlier study (Jarillo et al., 2001; Kagawa et al., 2001; Sakai et al., 2001; Suetsugu et al., 2016b; Sztatelman et al., 2016; Suetsugu and Wada, 2017), we proposed a model to explain the mechanisms underlying chloroplast movement under different BL intensities (Figure 7). Under LBL, phot1 and phot2 function redundantly to mediate chloroplast accumulation, with activated photoreceptors delivering the light signal to RPT2/NCH1 and JAC1. After a comprehensive analysis of *jac1* mutant, indicated that JAC1 mediates the chloroplast accumulation response and inhibits the chloroplast avoidance response under LBL conditions (Figure 7A). When the light intensity increased to 5 μmol m^−2^ s^−1^, the phot2-mediated avoidance response was activated in a manner that was independent of RPT2/NCH1-JAC1 signaling. However, under these conditions, JAC1 inhibition overrides this signal, resulting in normal chloroplast accumulation. However, *jac1* mutants do not possess this inhibition, resulting in an avoidance response under IBL (Figure 7B). As light intensity increases, the effect of NCH1 gradually weakens, resulting in an increase in the phot1-mediated accumulation signal, which is strong enough to overcome the inhibition of JAC1. As a result, the chloroplast avoidance response was present in the *phot2* mutant under HBL (Figure 7C).

## Data Availability Statement

The original contributions presented in the study are included in the article/Supplementary Material, further inquiries can be directed to the corresponding authors.

## Author Contributions

XZhao and XZhang designed the research and analyzed the results. JW, Y-pL, J-dZ, Y-xW, M-yY, and H-rY carried out the experiments. XZhang prepared the reagents and materials for the experiments. XZhang, JW, J-dZ, Y-pL, Q-yL, and KC organized and wrote the article. All authors have read and approved this manuscript.

## Funding

This study was supported by the National Natural Science Foundation of China (Grant numbers 31871419, 31870272, 32070262, and 31570294), National Key Research and Development Program of China (Grant number 2016YFD0101900), and Program for Innovative Research Team (in Science and Technology) in University of Henan Province (Grant number 21IRTSTHN019).

## Conflict of Interest

The authors declare that the research was conducted in the absence of any commercial or financial relationships that could be construed as a potential conflict of interest.

## Publisher's Note

All claims expressed in this article are solely those of the authors and do not necessarily represent those of their affiliated organizations, or those of the publisher, the editors and the reviewers. Any product that may be evaluated in this article, or claim that may be made by its manufacturer, is not guaranteed or endorsed by the publisher.
