# Supplementary material for: Phototropin 1 Mediates High-Intensity Blue Light-Induced Chloroplast Accumulation Response in a Root Phototropism 2-Dependent Manner in Arabidopsis phot2 Mutant Plants
Source: Front Plant Sci. 2021 Sep 27;12:704618. doi: 10.3389/fpls.2021.704618 (PMC8502927; doi:10.3389/fpls.2021.704618)
Supplement: Supplementary file 1 [file Data_Sheet_1.docx]

**Supplementary materials**

**
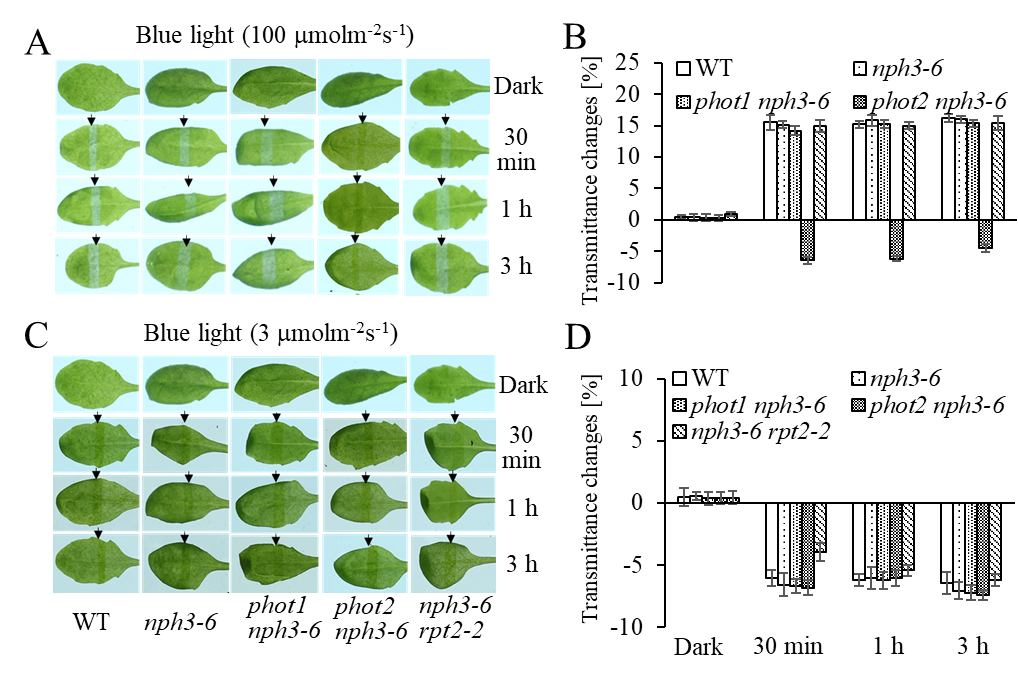
**

**Figure S1.** Chloroplast accumulation and avoidance are independent of NPH3 or cryptochromes. Detached rosette leaves of WT and mutant plants pretreated with 12 hours of dark were placed on a 0.8% agar medium, covered with a [light](C:/Users/zhujindong/AppData/Local/youdao/dict/Application/8.9.3.0/resultui/html/index.html#/javascript:;) [barrier](C:/Users/zhujindong/AppData/Local/youdao/dict/Application/8.9.3.0/resultui/html/index.html#/javascript:;) with a 2 mm wide [nonopaque](C:/Users/zhujindong/AppData/Local/youdao/dict/Application/8.9.3.0/resultui/html/index.html#/javascript:;) slit, and irradiated with continuous blue light (A, B 100 μmol m^−2^ s^−1^; C, D 3 μmol m^−2^ s^−1^) for 30 minutes, 1 hour, or 3 hours in a growth chamber. (B, D) Chloroplast photorelocation movement in the rosette leaves of WT, *nph3-6*, *phot1 nph3-6*, *phot2 nph3-6*, and *nph3-6 rpt2* plants. Light transmittance was measured in 21-day-old rosette leaves. Error bars represent the SD of 21 rosette leaves.


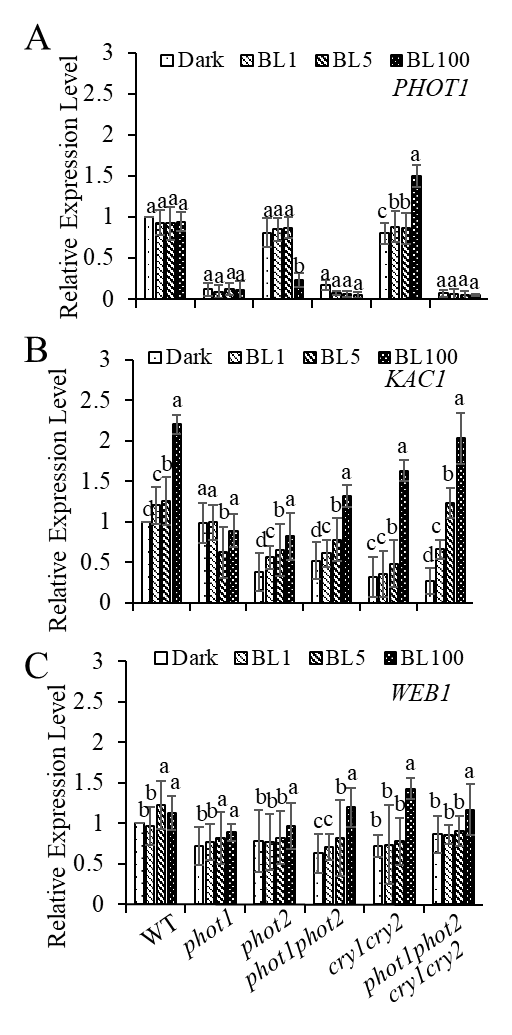


**Figure S2.** Quantitative RT-PCR analysis of (A) *PHOT1*, (B) *KAC1*, (C) *WEB1* in WT, *phot1*, *phot2*, *phot1 phot2*, *cry1 cry2* and *phot1 phot2 cry1 cry2* plants. Seedlings were irradiated with continuous blue light (1 μmol m^−2^ s^−1^, 5 μmol m^−2^ s^−1^, 100 μmol m^−2^ s^−1^) for 3 hours prior to expression quantification. Error bars represent the SD of three independent biological replicates (n = 3). A one-way ANOVA and Tukey’s test were performed to identify significant differences. Groups marked with different letters are significantly different (p < 0.05).

**Table S1** Primers of qPCR used in this study

| primer | sequence | primer | sequence |
| --- | --- | --- | --- |
| ACTIN2-F | AACCACTATGTTCTCAGGCATCG | ACTIN2-R | CCTGGACCTGCCTCATCATACT |
| PMI2-F | AGAGACGACGTGAACCAATC | PMI2-R | GACCTTACAGGTGAGTGAGAAG |
| KAC1-F | CTTGTGCTCTGACTTTCGTTTG | KAC1-R | CGAAGGGTTTGGAATAGGAGAA |
| KAC2-F | GGAAAGCTGAGGGAGATACAAG | KAC2-R | TCTGCAAGCCATGACCTAATC |
| PHOT1-F | AGCCTCACAGGAAAGATTCAC | PHOT1-R | AACGAGTTCCACTAGATGCAC |
| PHOT2-F | TGGGATGCAGGTTGAAGTTAG | PHOT2-R | ACAACCTCTGTGATGGAATCC |
| RPT2-F | CTTCCTTAGCCTCTGCAATCA | RPT2-R | ATCTCCGGGATCGGAATATTTG |
| JAC1-F | ATAGTAGCCCGATACCCGATAA | JAC1-R | TCCAACTGAAGCTCCTTTACTG |
| NCH1-F | CACGAATCCAAGTTCACCAATC | NCH1-R | GGGTTTAGCTTACCGAGTTTCT |
| CHUP1-F | GCCACAGGTGATCAGTCTAAC | CHUP1-R | GGTACTCTAGGAGGTCGTTTCT |
| WEB1-F | GAGGGTTGAAGAGATGGAACAA | WEB1-R | CCTCCTTGACAGAAGACAACTC |
